# Successful Venous Angioplasty of Superior Vena Cava Syndrome after Heart Transplantation

**DOI:** 10.1155/2014/490276

**Published:** 2014-08-03

**Authors:** Thomas Strecker, Iris Zimmermann, Marco Heinz, Johannes Rösch, Abbas Agaimy, Michael Weyand

**Affiliations:** ^1^Center of Cardiac Surgery, Friedrich-Alexander-University Erlangen-Nuremberg, Krankenhausstraße 12, 91054 Erlangen, Germany; ^2^Department of Radiology, Friedrich-Alexander-University Erlangen-Nuremberg, Erlangen, Germany; ^3^Institute of Pathology, Friedrich-Alexander-University Erlangen-Nuremberg, Erlangen, Germany

## Abstract

*Introduction*. For patients with terminal heart failure, heart transplantation (HTX) has become an established therapy. Before transplantation there are many repeated measurements with a pulmonary artery catheter (PAC) via the superior vena cava (SVC) necessary. After transplantation, endomyocardial biopsy (EMB) is recommended for routine surveillance of heart transplant rejection again through the SVC. *Case Presentation*. In this report, we present a HTX patient who developed a SVC syndrome as a possible complication of all these procedures via the SVC. This 35-year-old Caucasian male could be successfully treated by balloon dilatation/angioplasty. *Conclusion*. The SVC syndrome can lead to pressure increase in the venous system such as edema in the head and the upper part of the body and further serious complications like cerebral bleeding and ischemia, or respiratory problems. Balloon angioplasty and stent implantation are valid methods to treat stenoses of the SVC successfully.

## 1. Introduction 

For patients with terminal heart failure, heart transplantation (HTX) has become an established therapy [1, 2]. Before transplantation there are many repeated measurements with a pulmonary artery catheter (PAC) via the superior vena cava (SVC) necessary [3]. After transplantation, endomyocardial biopsy (EMB) is recommended for routine surveillance of heart transplant rejection again through the SVC [4].

## 2. Case Presentation 

A 35-year-old Caucasian male suffered from dilated cardiomyopathy (DCM) with severely impaired left ventricular function for 5 years (the ejection fraction (EF) was approximately 10%). Subsequently, he underwent mitral valve reconstruction and implantation of a biventricular cardioverter-defibrillator (ICD). One year later, he had an embolic insult with consecutive hemiplegia on the left side, from which he recovered well. He could be treated on an ambulatory level for quite a long time; however he had recurrent episodes of cardiac decompensation.

Currently, he was admitted again to our hospital with acute terminal cardiac insufficiency and we had to start the intravenous catecholamine therapy to recompensate this young patient. Mainly based on the results of the pulmonary artery catheter (PAC) which measured the cardiac output, the stroke volume, and intracardiac pressure, he was put on the waiting list for cardiac transplantation in the “high urgent” category. The PAC was inserted through the superior vena cava (SVC) into the right side of the heart and floated into the pulmonary artery. All these measurements must be repeated every 8 weeks for the reevaluation of the “high urgent” status. After more than one-year waiting period, a suitable donor organ was available and the heart transplantation (HTX) could be carried out successfully. Subsequently, endomyocardial biopsies (EMB) were performed to control the cardiac allograft rejection, because EMB still represents the gold standard for routine surveillance for HTX rejection.

**Figure 1:**
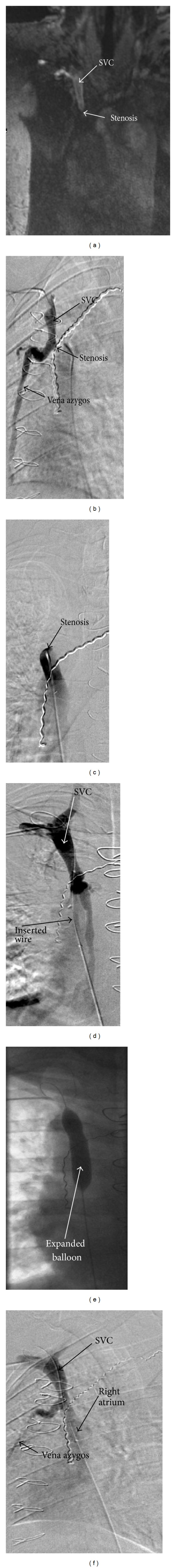
(a) Magnetic resonance tomography (MRT): breakup of the contrast medium in the superior vena cava (SVC) just before the right atrium. (b) Cavography depicted a subtotal stenosis of the SVC. Notably, the collateral contrast enhancement in the vena azygos. (c) Retrograde exposure of the SVC stenosis. (d) Successfully retrograde wire sounding of the SVC stenosis. (e) Effectively balloon-dilatation (maximum 10 mm diameter). (f) Postinterventional cavography revealed a stenosis-free flow/passage into the right atrium. Noteworthy, the clear decline of the collateral contrast enhancement in the vena azygos.

With increasing swelling in the neck which was associated with occasional swallowing difficulties, a significant increase in weight, and inability to perform further EMB via SVC, a magnetic resonance tomography (MRT) of the thorax was performed. This MRT revealed a subtotal stenosis of the SVC just before the intersection into the right atrium (Figure 1(a)). Furthermore, a closure of the vena anonyma was depicted. Thereupon, a cavography was carried out and the VCS stenosis was dilated and reopened via balloon angioplasty (Figures 1(b)–1(f)). Afterwards, it came for a clear improvement of the discomfort and the general medical situation of this HTX patient. Altogether, he lost approximately 25 kilograms of body weight in the following 3 months.

## 3. Discussion 

Stenoses of the SVC by frequent punctures through PAC investigations and EMB are a serious complication in patients suffering from cardiac insufficiency and HTX patients. Although obstructions of the SVC due to such cardiac or vessel interventions are often asymptomatic or their symptoms improve with time due to formation of decompressive collateral pathways to the right atrium [5], the SVC syndrome can lead to pressure increase in the venous system such as edema in the head and the upper part of the body and further serious complications like cerebral bleeding and ischemia, or respiratory problems. Balloon angioplasty and stent implantation are valid and reliable methods to treat stenoses of the SVC successfully [6]. It has become the first-line treatment of obstructions of the SCV and the success and complication rates are very promising [7].

## References

[1] Boyle A (2009). Current status of cardiac transplantation and mechanical circulatory support. *Current Heart Failure Reports*.

[2] Frazier OH (2010). Current status of cardiac transplantation and left ventricular assist devices. *Texas Heart Institute Journal*.

[3] Gidwani UK, Mohanty B, Chatterjee K (2013). The pulmonary artery catheter: a critical reappraisal. *Cardiology Clinics*.

[4] Strecker T, Rösch J, Weyand M, Agaimy A (2013). Endomyocardial biopsy for monitoring heart transplant patients: 11-years-experience at a german heart center. *International Journal of Clinical and Experimental Pathology*.

[5] Kogon BE, Plattner C, Jennings S, Lyle T, McConnell M, Book WM (2008). Cyanosis produced by superior vena caval stenosis. *Annals of Thoracic Surgery*.

[6] Sherry CS, Diamond NG, Meyers TP, Martin RL (1986). Successful treatment of superior vena cava syndrome by venous angioplasty. *The American Journal of Roentgenology*.

[7] Warner P, Uberoi R (2013). Superior vena cava stenting in the 21st century. *Postgraduate Medical Journal*.

